# Supplementary material for: Prenatal Cadmium Exposure Alters Proliferation in Mouse CD4+ T Cells via LncRNA Snhg7
Source: Front Immunol. 2022 Jan 11;12:720635. doi: 10.3389/fimmu.2021.720635 (PMC8786704; doi:10.3389/fimmu.2021.720635)
Supplement: Supplementary file 1 [file Image_1.pdf]

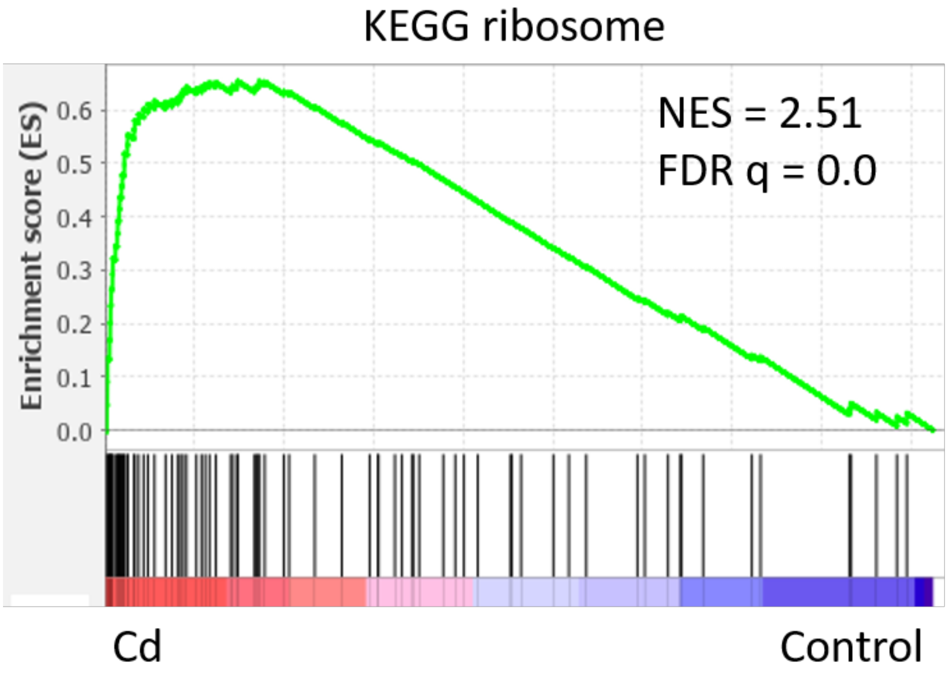

Figure S1. Ribosome-associated genes are upregulated in T cells of Cd-exposed offspring. Gene set enrichment analysis (GSEA) of expressed genes in in vitro activated T cells from offspring against MSigDB gene set “KEGG ribosome”; genes sorted by FC of expression (parental Cd exposed/control) from high (red) to low (blue).

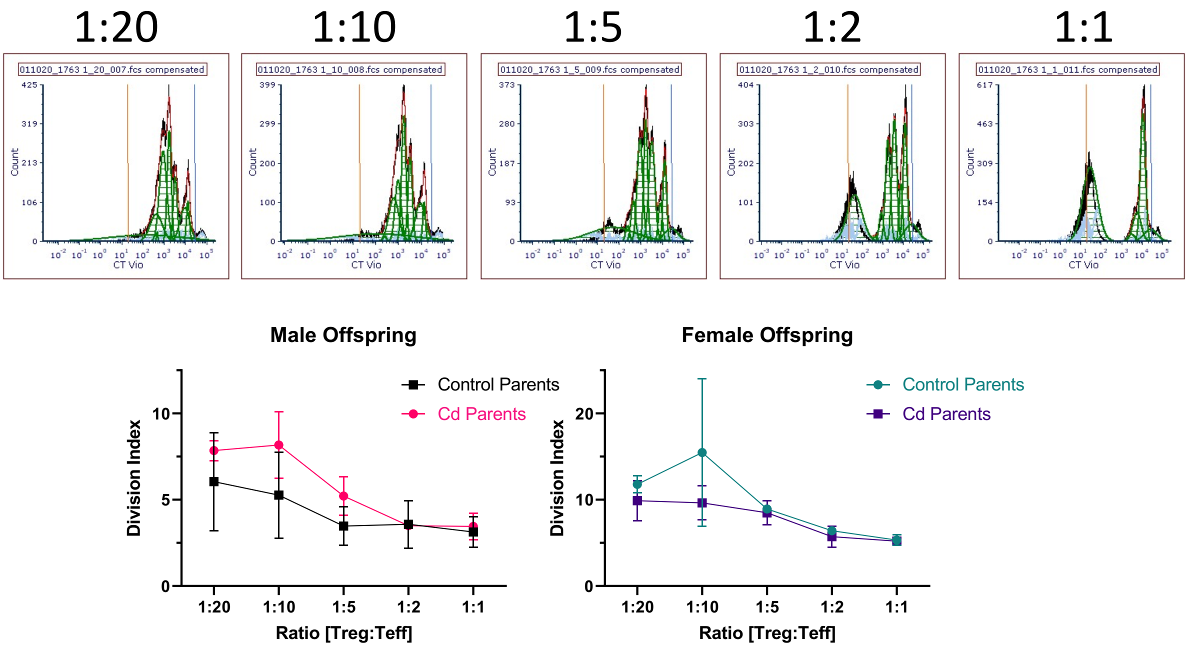

Figure S2. Suppression ability of Tregs from Cd offspring is unaltered. The suppression ability of $CD4^{+}CD25^{+}$ T regulatory cells from control and Cd offspring were assessed at 5 days.

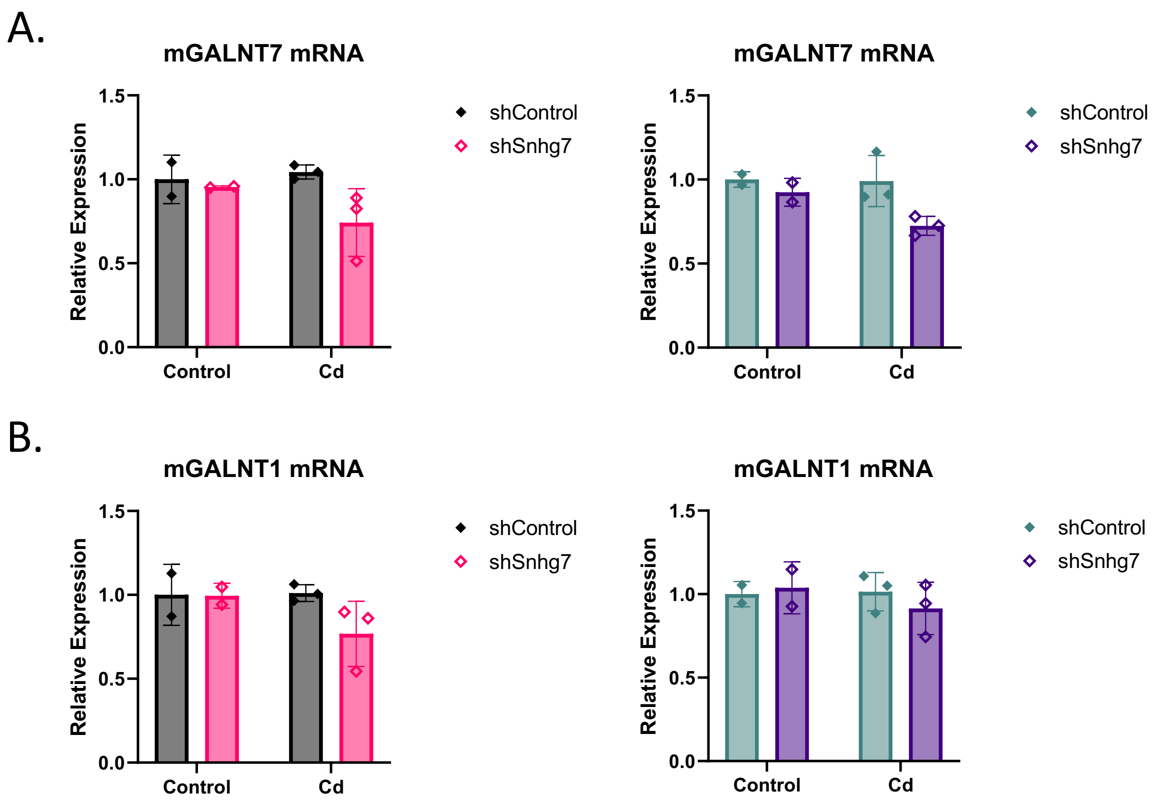

Figure S3. Knockdown of lncSnhg7 in primary CD4$^+$ T cells does not alter GALNT7 or GALNT1 mRNA expression. Primary CD4$^+$ T cells were isolated from mice splenocytes and labeled with Cell Trace Violet. T cells were stimulated with anti-CD3/CD28 magnetic beads in the presence of control- or lncSnhg7-targeted lentivirus for 18 h. Media was replaced, and stimulation proceeded for 72 h total before analysis. A) GALNT7 and B) GALNT1 expression were assessed by qPCR.

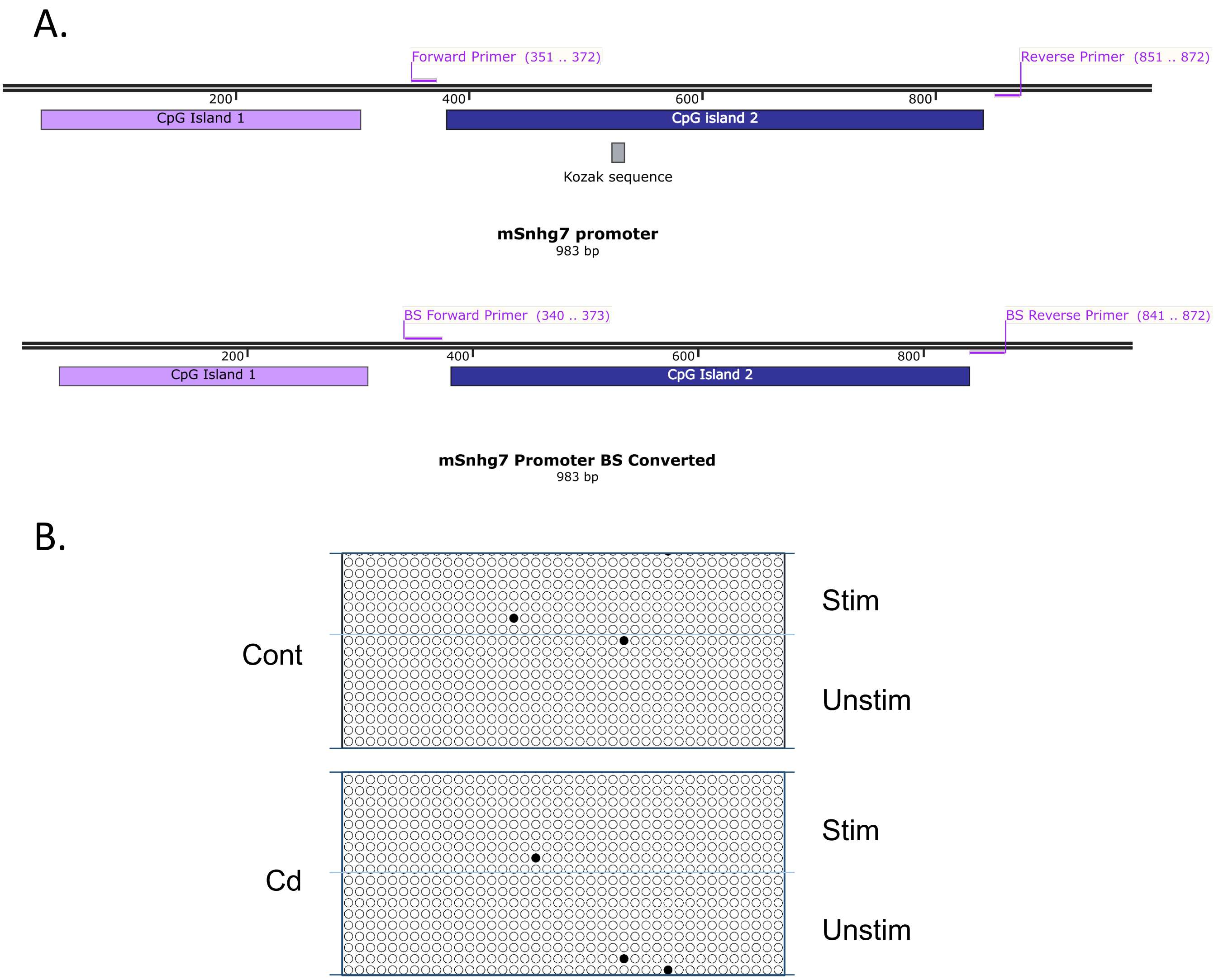

Figure S4. Prenatal Cd exposure does not alter lncSnhg7 promoter methylation in CD4+ T cells. A) Regions of PCR amplification in the unconverted and bisulfite-converted DNA; B) Representative data of methylation of CpG sites in control (top) and Cd (bottom) offspring.

GS	logFC	FDR
Map2k3os	2.98	1.11E-11
AI506816	2.94	2.39E-44
Snhg4	2.75	3.13E-72
Dancr	1.92	8.82E-15
Snhg20	1.81	2.88E-13
Rab26os	1.71	5.60E-06
Dnmt3aos	1.67	3.20E-06
Snhg7	1.35	2.27E-13
Pvt1	1.22	3.05E-19
AI662270	1.2	4.57E-51
Smarca5-ps	1.17	1.46E-29
Mir17hg	1.14	1.40E-26
Zfp783	1.14	1.21E-12
Slain1os	-1.1	3.58E-06
H2-K2	-1.2	1.24E-31
Tbc1d22bos	-1.25	1.83E-06
BC043934	-1.3	1.06E-14
Rapgef4os2	-1.39	0.000348
Zbtb11os1	-1.39	2.38E-07
Rbm3os	-1.41	4.94E-07
Mir22hg	-1.63	7.73E-10
Peg13	-1.82	6.23E-105
AW011738	-1.94	1.56E-43

Table S1: List of lncRNAs differentially expressed between unstimulated and stimulated CD4+ T cells.
